# Heat Stress Altered the Vaginal Microbiome and Metabolome in Rabbits

**DOI:** 10.3389/fmicb.2022.813622

**Published:** 2022-04-14

**Authors:** Yu Shi, Lipeng Tang, Xue Bai, Kun Du, Haoding Wang, Xianbo Jia, Songjia Lai

**Affiliations:** Farm Animal Genetic Resources Exploration and Innovation Key Laboratory Province, Sichuan Agricultural University, Chengdu, China

**Keywords:** heat stress, vagina, microbiome, metabolome, rabbit

## Abstract

Heat stress can have an impact on parental gamete maturation and reproduction functions. According to current research, the microbial composition of the vaginal cavity is species specific. Pregnancy, menstruation, and genital diseases have been linked to the dynamics of vaginal ecology. In this study, we characterized the vaginal microbiota and metabolites after heat stress. At the phylum level, the rabbit’s vaginal microbial composition of rabbit showed high similarity with that of humans. In the Heat group, the relative abundance of the dominant microbiota *Actinobacteria*, *Bacteroidetes*, and *Proteobacteria* increased, while the relative abundance of *Firmicutes* decreased. Furthermore, heat stress significantly increased the relative abundance of *W5053*, *Helcococcus*, *Thiopseudomonas*, *ldiomaarina*, *atopostipes*, and *facklamia*, whereas the relative abundance of 12 genera significantly decreased, including *Streptococcus*, *UCG-005*, *Alistipes*, *[Eubacterium]_xylanophilum_group*, *Comamonas*, *RB41*, *Fastidiosipila*, *Intestinimonas*, *Arthrobacter*, *Lactobacillus*, *Leucobacter*, and *Family_xlll_AD3011_group.* Besides, the relative concentrations of 158 metabolites differed significantly between the Heat and Control groups. Among them, the endocrine hormone estradiol (E_2_) increased in the Heat group and was positively associated with a number of metabolites such as linolelaidic acid (C18:2N6T), N-acetylsphingosine, N-oleoyl glycine, trans-petroselinic acid, syringic acid, 2-(1-adamantyl)-1-morpholinoethan-1-one, 5-OxoETE, and 16-heptadecyne-1,2,4-triol. Further, the majority of the differential metabolites were enriched in steroid biosynthesis and endocrine and other factor-regulated calcium reabsorption pathways, reflecting that heat stress may affect calcium metabolism, hormone-induced signaling, and endocrine balance of vaginal ecology. These findings provide a comprehensive depiction of rabbit vaginal ecology and reveal the effects of heat stress on the vagina *via* the analysis of vaginal microbiome and metabolome, which may provide a new thought for low female fertility under heat stress.

## Introduction

Heat stress has been proved to be detrimental to the physiological and metabolic activities of animals (Bagath et al., 2019). Rabbits are known to be sensitive to high temperatures, which might be due to the shortage of sweat glands that effectively dissipate body heat (Oshima et al., 2011) and the presence of thick fur that prevents heat loss (Marai et al., 2001). When the ambient temperature reaches 31°C or above, rabbits show a significant decrease in hemoglobin concentration, red blood cells, and packed cell volume. On the other hand, white blood cells, lymphocytes, creatinine, urea, and aspartate transaminase increase, implying that heat stress strengthens oxidative stress and impairs physiological functions (Mutwedu et al., 2021). Heat stress is also known to have a detrimental effect on the production performance of female rabbits. For example, compared to female rabbits living in appropriate temperatures, those suffering from heat stress produce fewer litters and fewer live-born kits (Marco-Jiménez et al., 2013). In bovine, a negative relationship has been observed between the conception rate and the rectal temperature (Ulberg and Burfening, 1967). The temperature of the genital tract including the vagina and cervix was elevated as the ambient temperature rose (Nabenishi et al., 2011; El-Sheikh Ali et al., 2020), indicating that heat stress could potentially cause a disturbance in the vaginal microenvironment.

Nowadays, considerable efforts have been made to assess the role of microorganisms and the metabolome of the reproductive tract in female health. In clinical cases, the shift in the vaginal microbiota from dominant Lactobacillus to a polymicrobial microbiota is usually diagnosed as bacterial vaginosis (BV) (Onderdonk et al., 2016). The microbial composition of the vagina is believed to be dynamic in a transitional period, such as pregnancy or menstruation (Hickey et al., 2013; Gupta et al., 2020). Previous studies have suggested that acute heat stress could cause intestinal metabolism disorders and microbiota changes in mice (Wen et al., 2021), pigs (Xiong et al., 2020), and poultry (Zhu et al., 2019). Long-term heat stress not only leads to a disorder of the estrous cycle but also alters the vaginal bacterial communities in rats (An et al., 2021).

Although the vaginal microbiome might provide evidence for female clinical diagnosis, the effect of heat stress on the ecological community and associated metabolites in the vagina has not been elucidated. The objective of this study is to reveal the vaginal microenvironment of rabbits and to explore the effects of heat stress on the vaginal microbiota and metabolites, along with the mechanisms affecting the vaginal health of rabbits.

## Materials and Methods

### Animal Manipulation and Sample Collection

In order to avoid the influence of mating or reproduction on the vagina ecology (Lewis et al., 2017; Gupta et al., 2020), an experimental group of 48 healthy and virgin female New Zealand rabbits aged 7∼8 months were randomly divided into two groups, i.e., Heat group and Control group, hosed in separate rooms. The rabbits were caged separately but raised together within each group and had free access to water and food. To synchronize the estrous cycle, all female rabbits used were injected intramuscularly with 70∼80 IU of pregnant mare serum gonadotrophin (PMSG, Ningbo Second Hormone Factory, Zhejiang, China). After a 3-week normal feeding scheme, a heat-treated program was conducted in the Heat group from 9:00 a.m. to 5:00 p.m. each day and continued for 15 days. In contrast, no extra treatment was provided to the Control group, considering that the climate in April in Southwest China is relatively cool, with an average temperature of 23°C. The daily temperature and humidity were measured using a thermo-hygrometer (Delixi, Yueqing, China), and the temperature-humidity index (THI) was calculated using the formula:


(1) THI=T-[(0.31-0.31⁢RH)⁢(T-14.4)]

**RH = relative humidity/100; T = ambient temperature**

During the experiment, the Control group was hosted at a THI of 23.36 ± 1.86, and the Heat group at a THI of 30.04 ± 0.88. After half a month of the heat-treated program, several sterile cotton swabs were inserted into the rabbits’ vagina up to 3∼4 cm and rotated 1∼2 times to collect the vaginal content. The swabs from one individual were transferred to two sterile 15-ml centrifuge tubes (one sample was used for 16S DNA analysis and the other for the metabolome analysis) and stored at −80°C. On the same day, a blood sample of each rabbit was collected from the ear vein. A total of 16 individuals (8 from the Heat group and 8 from the Control group) were randomly selected from the experimental group for the following analysis.

### Measurement of SOD, T-AOC, CAT, MDA, E2, and P4

The concentrations of several related oxidative biomarkers including plasma superoxide dismutase (SOD, DRE-R3047c), total antioxidant capacity (T-AOC, DRE-R3098c), catalase (CAT, DRE-R3050c), malondialdehyde (MDA, DRE-R3190c), hormone estradiol (E_2_, DRE-R0447c), and progesterone (P_4_, DRE-R5802c) were determined following the protocol for ELISA Kits (R&D Systems, Minneapolis, MN, United States). The assay sensitivities were < 1.0 U/ml, 0.1 U/ml, 1.0 U/ml, 0.1 nmol/ml, 1.0 pg/ml, and 0.1 ng/ml, respectively. The coefficients of variation inter-assay and intra-assay were all less than 15%.

### DNA Extraction and Sequencing

The DNA of the vaginal samples was extracted using the CTAB method. The purity and concentration of the DNA were assessed using agarose gel electrophoresis. The DNA was then diluted to 1 ng/μl with sterile water. Barcode-specific primers 515F-806R were utilized to amplify the V4 region of the 16S rRNA gene. Purified PCR products were then recovered. The PCR products of different samples were mixed equally and then used to construct a DNA library using the TruSeq^®^ DNA PCR-Free Sample Preparation Kit (Illumina, San Diego, CA, United States). The quality of the library was evaluated using a Qubit @ 2.0 Fluorometer (Thermo Fisher Scientific, Waltham, MA, United States) and Q-PCR. The DNA sequencing was finally sequenced using the Illumina NovaSeq 6000.

### 16S rDNA Sequencing Process and Data Analysis

Raw reads of each sample were extracted based on their barcode sequence and then subsequently spliced using FLASH (V1.2.7)^1^ (Magoč and Salzberg, 2011). Then raw tags were filtered following the protocol (Caporaso et al., 2010) to produce clean tags. Effective tags were obtained after the chimeric sequences were removed. Sequences with 97% identity were clustered into the same operational taxonomic units (OTUs) using UPARSE v7.0.1001^2^, and the sequence with the highest frequency was selected as the representative OTUs. Annotation of the OTUs was conducted using the Mothur method and SILVA138^3^ (Edgar et al., 2011) based on the SSUrRNA database (Quast et al., 2013) with a confidence threshold of 0.8. The α- and β-diversities were then calculated using the QIIME software (Version 1.9.1) and consequently analyzed using the *t*-test method. Principal coordinate analysis (PCoA) analysis was displayed by the WGCNA package (Langfelder and Horvath, 2008), stat packages, and ggplot2 package (Wickham, 2009) in R software. The function prediction was conducted using Tax4Fun (Aßhauer et al., 2015).

### Ultra-High-Performance-Liquid-Chromatography-MS/MS Analysis of Vaginal Metabolomics

The swabs of each rabbit in the EP tubes were resuspended with prechilled 80% methanol by well vortex. The samples were then melted on ice and then whirled for 30 s. After the sonification for 6 min, the samples were centrifuged at 5,000 rpm at 4°C for 1 min. The supernatant was freeze-dried and dissolved with 10% methanol for further analysis. The detection was carried out using a Vanquish UHPLC system (Thermo Fisher Scientific, Bremen, Germany) coupled with an Orbitrap Q Exactive HF-X mass spectrometer (Thermo Fisher Scientific, Germany) in Novogene Co., Ltd. (Beijing, China). The samples were injected onto a Hypersil Gold column (100 × 2.1 mm, 1.9 μm) using a 17-min linear gradient at a flow rate of 0.2 ml/min. Eluent A (0.1% FA in water) and eluent B (methanol) were used as the eluents for the positive polarity mode. The eluents used for the negative polarity mode were eluent A (5 mM ammonium acetate, pH = 9.0) and eluent B (methanol). The solvent gradient was set as follows: 2% B, 1.5 min; 2–100% B, 12.0 min; 100% B, 14.0 min; 100–2% B, 14.1 min; 2% B, 17 min. The Q Exactive HF mass spectrometer was operated in positive/negative polarity mode with spray voltage of 3.2 kV, capillary temperature of 320°C, sheath gas flow rate of 40 arb, aux gas flow rate of 10 arb, funnel RF level of 40, and aux gas heater temperature of 350°C.

### UHPLC-MS/MS Data Processing and Metabolite Identification

The raw data files generated by UHPLC-MS/MS were processed using the Compound Discoverer 3.1 (CD3.1, Thermo Fisher Scientific) to perform peak alignment, peak picking, and quantitation for each metabolite. The main parameters were set as follows: retention time tolerance, 0.2 min; actual mass tolerance, 5 ppm; signal intensity tolerance, 30%; and signal/noise ratio, 3. Next, the peak intensities were normalized to the total spectral intensity. The normalized data were used to predict the molecular formula based on additive ions, molecular ion peaks, and fragment ions. Peaks were then matched with mzCloud^4^, mzVault, and MassList databases to obtain the accurate qualitative and relative quantitative results. Statistical analyses were performed using the statistical software R (R version R-3.4.3), Python (Python 2.7.6 version), and CentOS (CentOS release 6.6). In the event where data were not normally distributed, normal transformations were attempted using the area normalization method.

### UHPLC-MS/MS Data Analysis

The metabolites were annotated using the Kyoto Encyclopedia of Genes and Genomes (KEGG) database^5^, HMDB database^6^, and LIPIDMaps database^7^. Partial least square discriminant analysis (PLS-DA) was performed at metaX (Wen et al., 2017). Univariate analysis (*t*-test) was used to calculate the statistical significance (*P*-value). The metabolites with VIP > 1 and *P*-value < 0.05 and fold change ≥ 2 or fold change ≤ 0.5 were considered to be differential metabolites. The volcano plot was used to filter metabolites of interest which are based on log_2_ (FoldChange) and -log_10_(*P*-value) of metabolites by ggplot2 in R. For clustering heat maps, the data were normalized using z-scores of the intensity areas of differential metabolites and were plotted by Pheatmap package in R. The correlation between differential metabolites was analyzed by cor () in R (method = Pearson). A *P*-value < 0.05 was considered as statistically significant. The functions and metabolic pathways of metabolites with significant difference were studied using the KEGG database, and when *P*-value < 0.05, metabolic pathways were considered as statistically significant enrichment. All diagrams were plotted using R software.

## Results

### Heat Stress Effects on Oxidative Stress Biomarkers and Hormone of Serum

**FIGURE 1:**
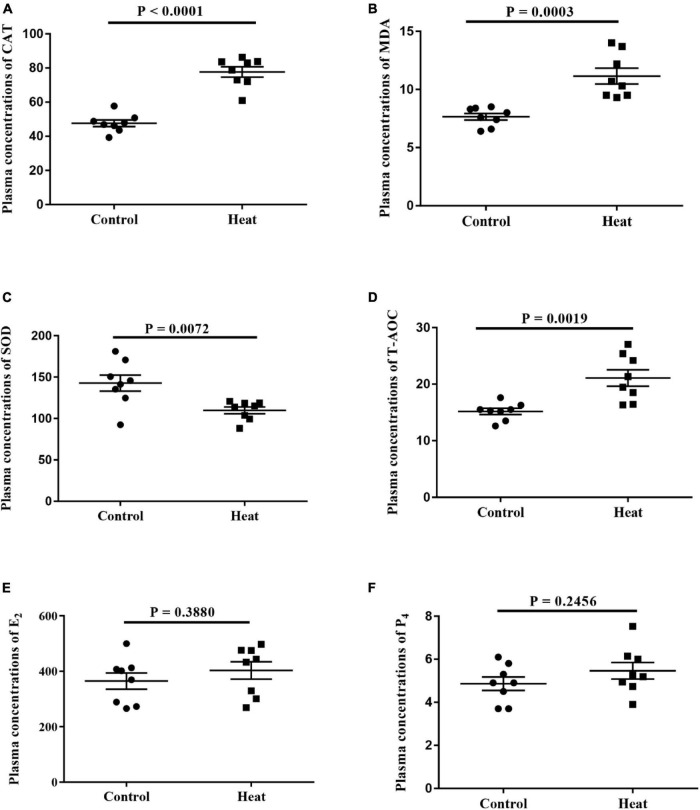
**(A–D)** The levels of oxidative stress-related biomarkers including superoxide dismutase (SOD), total antioxidant capacity (T-AOC), catalase (CAT) and malondialdehyde (MDA) in the serum are detected, respectively. **(E,F)** The levels of estrogen (E2) and progesterone (P4) in the serum are detected. A *P* < 0.05 indicates statistically significant difference (*t*-test). Error bar indicates the standard error of the mean.

The levels of oxidative stress-related biomarkers were determined to assess the model of heat stress and to determine if heat stress could disturb hormone secretion in rabbits. This included the biomarkers SOD, T-AOC, CAT, and MDA, and estrous cycle-related hormones E_2_ and P_4_ in serum. As shown in Figure 1, heat stress significantly increased the levels of T-AOC, CAT, and MDA, while decreasing the SOD level. This indicates the efficiency of the heat stress program and the heat stress-induced general oxidative stress in female rabbits. No significant differences were observed in the E_2_ and P_4_ levels between the Heat and Control groups.

### Diversities and Richness of Rabbit Vagina

**FIGURE 2:**
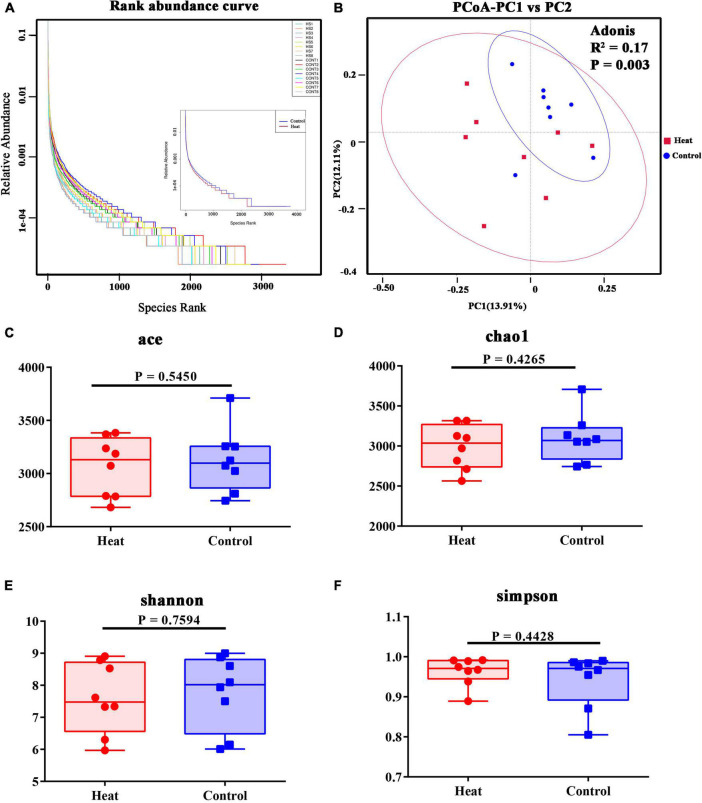
Diversities of vaginal microbiota of rabbit. **(A)** The rank abundance curve of 16 rabbits, and in the right, HS1–HS8 indicate the eight individual animals in the Heat group, and CONT1–CONT8 indicate the eight individual animals in the Control group. The right part of the diagram displays the average species rank of the two groups. **(B)** PCoA analysis and Adonis analysis of microbial communities. R2 indicates the explanation degree of the grouping method for the difference between samples. The greater R2, the higher the explanation degree of the grouping method for the difference. *P* < 0.05 indicates the reliability of this inspection is high. **(C–F)** The alpha diversity indices include Chao1, ACE, Shannon, and Simpson. The statistical differences were calculated using the *t*-test. *P* < 0.05 indicates significant difference.

An average of 81,207 raw reads was generated. An average of 62,722 effective tags was acquired after removing the low-quality and short-length or chimeric tags (Table 1). The abundance and evenness of microbiota in each group are shown in Figure 2A. Alpha diversity indices including Chao1, Ace, Shannon, and Simpson were used to assess the ecological diversity within each group (Figures 2C–F). No significant differences were observed in the species richness or diversities between the Heat and Control groups. The PCoA and Adonis analysis indicated that heat stress significantly changed the microbial composition of the rabbit vagina (Figure 2B).

**TABLE 1:** Data processing, statistics, and quality control.

Sample name	Raw PE^a^	Combined^b^	Qualified^c^	Nochime^d^	Q20^e^	Q30^f^	Effective^g^ (%)
HS1	86,264	85,810	85,457	69,740	99.18	97.7	80.84
HS5	74,335	73,093	72,452	61,409	99.2	97.73	82.61
HS2	83,941	83,228	82,626	63,216	99.16	97.63	75.31
HS3	80,814	80,109	79,565	60,872	99.13	97.59	75.32
HS4	84,449	83,680	83,160	64,486	99.14	97.64	76.36
HS6	76,780	76,491	76,148	60,696	99.06	97.44	79.05
HS7	78,530	77,982	77,560	60,444	99.09	97.53	76.97
HS8	87,677	87,504	87,170	69,280	99.14	97.57	79.02
CONT1	85,876	84,392	83,604	64,125	99.09	97.45	74.67
CONT2	78,492	77,586	77,046	61,280	99	97.25	78.07
CONT3	79,467	79,126	78,832	61,317	99.06	97.37	77.16
CONT4	79,122	77,913	77,087	60,291	99.09	97.45	76.2
CONT5	78,845	78,394	78,061	60,877	99	97.26	77.21
CONT6	85,473	84,129	83,398	63,499	99.11	97.53	74.29
CONT7	80,470	79,012	78,231	61,885	99.13	97.55	76.9
CONT8	78,775	77,141	76,418	60,128	99.09	97.37	76.33
Average	81,207	80,349	79,801	62,722	99	98	77

*^a^Raw pair-end reads. Number of combined tags. ^c^Number of remaining tags after removing low-quality and short sequences. ^d^Effective tags after removing chimera. ^e^The percentage of bases with base quality value greater than 20 (sequencing error rater less than 1%) in effective Tags. ^f^The percentage of bases with base quality value greater than 30 (sequencing error rater less than 0.1%) in effective Tags. ^g^Percentage of the number of effective Tags to the number of raw pair-end reads.*

### Taxonomic Composition of Vaginal Bacterial Communities

**FIGURE 3:**
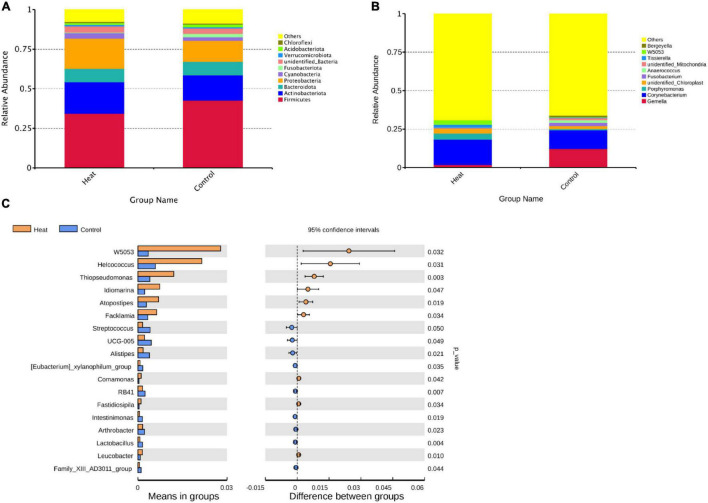
The effects of heat stress on vaginal microbial composition of rabbit. **(A)** The average relative abundance of the top 10 abundant vaginal microbiota at the phylum level. **(B)** The average relative abundance of the top 10 abundant vaginal microbiota at the genus level. “Others” represents the proportion of microbiota unannotated or with low abundance at the phylum and genus levels, respectively. **(C)** Genera with significant difference in relative abundance between Heat and Control groups. *p*-value < 0.05 indicates that the relative abundance microbiota differ significantly between the Heat and Control groups.

Taxonomic profiling indicated diverse microbiota between the Heat and Control groups. As presented in Figure 3A, the microenvironment of the rabbit vagina was dominated by three phyla, i.e., *Firmicutes* (averagely 34.4% in the Heat group and 42.7% in the Control group), *Actinobacteriota* (averagely 19.9% in the Heat group and 16.0% in the Control group), and *Proteobacteria* (averagely 19.1% in the Heat group and 13.3% in Control group) (Figure 3A). Moreover, additional obvious changes in the vaginal microbiota of the Heat and Control groups were observed at the genus level (Figure 3B). The most dramatic change observed was the abundance of *Gemella*, *Corynebacterium*, and *Porphyromona* between the two groups, wherein the average relative abundance of *Corynebacterium* and *Porphyromonas* increased from 12.1% (Control group) to 16.5% (Heat group) and from 0.8 to 3.9%, respectively. On the other hand, *Gemella* decreased from 12.2% (Control group) to 1.8% (Heat group). The relative abundance of the dominant microbiota for each individual rabbit is obtained in Supplementary Table 1. On a global scale, the abundance of 18 genera was significantly different between the Heat and Control groups. Heat stress dramatically increased the abundance of *W5053*, *Helcococcus*, *Thiopseudomonas*, *ldiomaarina*, *Atopostipes*, *Facklamia*, while decreasing *Streptococcus*, *UCG-005*, *Alistipes*, *[Eubacterium]_xylanophilum_group, Comamonas*, *RB41*, *Fastidiosipila*, *Intestinimonas*, *Arthrobacter*, *Lactobacillus*, *Leucobacter*, and *Family_xlll_AD3011_group* in the rabbit vagina (Figure 3C).

**FIGURE 4:**
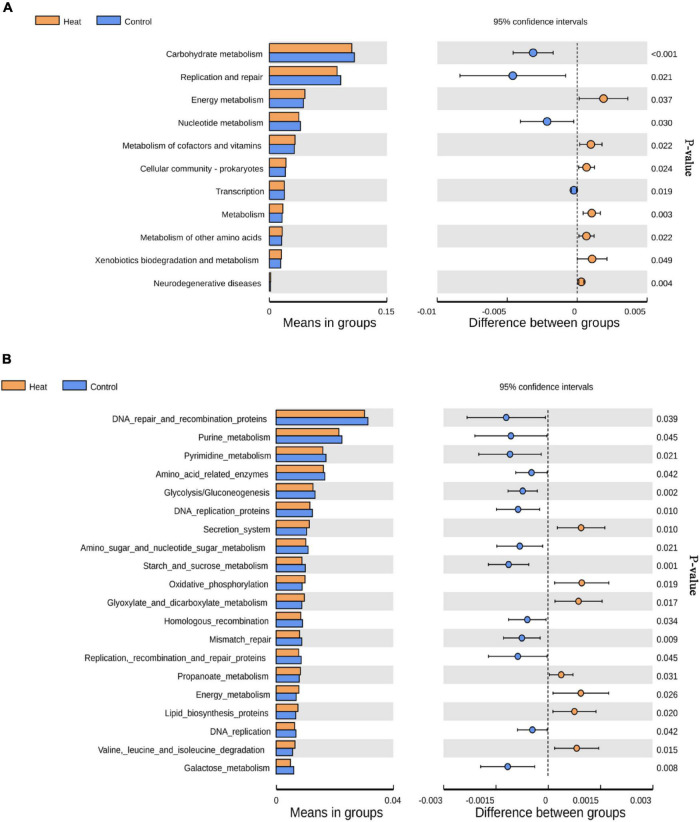
KEGG pathways affected by heat stress. **(A)** The level 2 KEGG pathways with statistically significant difference. **(B)** The top 20 level 3 KEGG pathways with the most significant difference between the Heat and Control groups. *P* < 0.05 indicates that the pathway is differentially enriched by the Heat and Control groups.

To determine the potential impact of the altered community in vagina health or function, KEGG pathways for 16S rDNA sequencing data were predicted using the Tax4Fun software. As presented in Figure 4, 11 KEGG pathways at level 2 were significantly different and included carbohydrate metabolism, replication and repair, energy metabolism, nucleotide metabolism, metabolism of cofactors and vitamins, cellular community-prokaryotes, transcription, metabolism, metabolism of other amino acids, xenobiotics biodegradation and metabolism, and neurodegenerative diseases (Figure 4A). At level 3 of the KEGG pathway, the top 20 abundant pathways with statistical differences were identified, as shown in Figure 4B.

### Alterations of Metabolism in Rabbit Vagina

**FIGURE 5:**
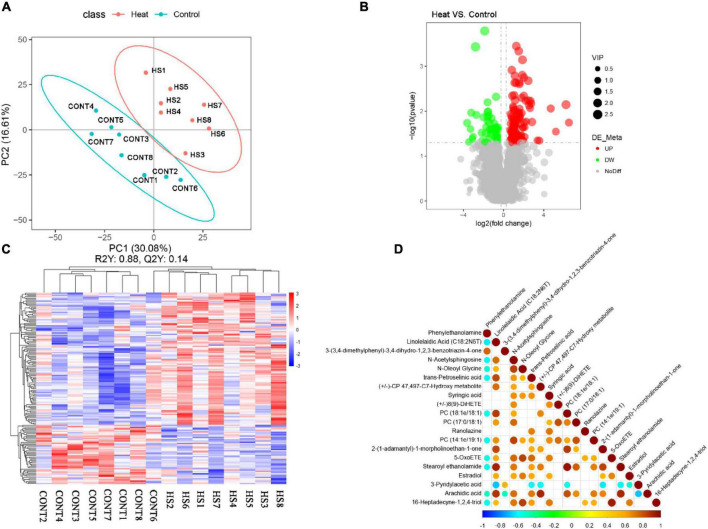
Heat stress affected the vaginal metabolome of rabbit. **(A)** PLS-DA analysis of metabolites. Numbers and points in red and blue represent samples of the Control and Heat groups, respectively. **(B)** Volcano plot of metabolites. The abscissa represents the change in the relative concentrations of metabolites in different groups (log2fc), and the ordinate represents the difference significance level [–log10 (*p*-value)]. Each point in the figure represents a metabolite; the size of the point represents the VIP value. Red and green points represent significantly up- and downregulated metabolites, respectively. Moreover, the gray point represents metabolites with no significant difference between Heat and Control groups. **(C)** Hierarchical clustering of metabolites with significant differences. Red and blue represent higher and lower concentrations of metabolites, respectively (*p* < 0.05). **(D)** Correlation analysis of the top 20 of significantly differential metabolites. Red and blue points represent the significantly positive and negative correlations, respectively (*p* < 0.05). The size of the point represents the absolute value of the correlation coefficient.

By using the UHPLC-MS/MS technology, a total of 2,033 metabolites were detected in the vaginal samples (Supplementary Table 2). The annotations for all metabolites were summarized (Supplementary Figure 1). The partial least square discrimination analysis (PLS-DA) indicated an obvious distinction between the Heat and Control groups (R2Y = 0.88, Q2 = 0.14, Figure 5A). The metabolites with VIP > 1 and *p*-value < 0.05 and fold change ≥ 2 or fold change ≤ 0.5 were considered to be differential metabolites (Figure 5B). The tendencies of variation of these metabolites are displayed in Figure 5C. Among these altered metabolites, there was a significant increase in the relative concentration of 109 metabolites in the Heat group, while 49 showed a notable decrease. The top 20 metabolites with the most significant changes are demonstrated in Table 2. Among them, the relative concentrations of 3 metabolites decreased in the Heat group, including phenylethanolamine, 3-(3,4-dimethylphenyl)-3,4-dihydro-1,2,3-benzotriazin-4-one, and 3-pyridylacetic acid. There was, however, an increase in the relative concentrations of 17 metabolites increased.

**TABLE 2:** The top 20 differential metabolites.

Name	Formula	*p*-value	VIP	Up. down
Phenylethanolamine	C8 H11 N O	0.000164258	2.482239648	Down
Linolelaidic acid (C18:2N6T)	C18 H32 O2	0.000356087	2.093793992	Up
3-(3,4-Dimethylphenyl)-3,4-dihydro-1,2,3-benzotriazin-4-one	C15 H13 N3 O	0.00037033	2.621861558	Down
N-Acetylsphingosine	C20 H39 N O3	0.000459944	2.078521958	Up
N-Oleoyl glycine	C20 H37 N O3	0.000721261	2.196232827	Up
Trans-Petroselinic acid	C18 H34 O2	0.001213849	2.035221701	Up
(+/–)-CP 47,497-C7-Hydroxy metabolite	C21 H34 O3	0.001570509	1.94299029	Up
Syringic acid	C9 H10 O5	0.001629517	2.021804394	Up
(+/–)8(9)-DiHETE	C20 H32 O4	0.001779308	1.943390378	Up
PC (18:1e/18:1)	C44 H86 N O7 P	0.002098441	2.076605267	Up
PC (17:0/18:1)	C43 H84 N O8 P	0.00228603	1.908144082	Up
Ranolazine	C24 H33 N3 O4	0.002326609	1.947152478	Up
PC (14:1e/19:1)	C41 H80 N O7 P	0.002343975	2.04324016	Up
2-(1-Adamantyl)-1-morpholinoethan-1-one	C16 H25 N O2	0.002490665	1.924706566	Up
5-OxoETE	C20 H30 O3	0.004402968	1.867548705	Up
Stearoyl ethanolamide	C20 H41 N O2	0.004545477	1.781861087	Up
Estradiol	C18 H24 O2	0.00457184	1.937636073	Up
3-Pyridylacetic acid	C7 H7 N O2	0.004791349	1.899803463	Down
Arachidic acid	C20 H40 O2	0.005051629	1.78259421	Up
16-Heptadecyne-1,2,4-triol	C17 H32 O3	0.005706042	2.052893582	Up

*Basing on the p-value in an ascending order, the top 20 metabolites with the most significant difference were shown. P-value: significance valued by t-test. VIP: the contribution of the metabolites to the grouping. Up.Down: Up indicates that compared with the Control group, and the relative concentration of metabolites increased in the Heat group. Down indicates that the relative concentration of metabolites deceased in the Heat group.*

Metabolites within the same ecology are correlated and impact each other. Thus, the correlation analysis of the top 20 metabolites with the most significance is presented in Figure 5D. Among them, estradiol demonstrated a positive association with linolelaidic acid (C18:2N6T), N-acetylsphingosine, N-oleoyl glycine, trans-petroselinic acid, syringic acid, 2-(1-adamantyl)-1-morpholinoethan-1-one, 5-OxoETE, and 16-heptadecyne-1,2,4-triol. To determine the potential roles of the differential metabolites in the biological functions and health of rabbit vagina, the metabolic pathways enriched by differential metabolites were identified using the KEGG database (Table 3). Moreover, it showed that differential metabolites were significantly enriched by steroid biosynthesis and endocrine and other factor-regulated calcium reabsorption.

**TABLE 3:** KEGG pathways affected by heat stress.

Name	*p*-value
Endocrine and other factor-regulated calcium reabsorption	0.0028
Steroid biosynthesis	0.008126

## Discussion

With the rise in global warming, heat stress has been a severe challenge affecting the reproduction function of female animals and human beings. Previous studies have indicated that heat stress affects the quality of parental gametes, subsequently leading to a failure in fertilization and embryo development *in vivo* or *in vitro* (Al-Katanani et al., 2002; Ozawa et al., 2002; Aroyo et al., 2007; Paul et al., 2008; Hendricks et al., 2009). Previous studies have also confirmed that heat stress can disrupt ovarian signaling (Dickson et al., 2018), impair estradiol synthesis of granulosa cells (Li H. et al., 2017), and affect ovarian hormone secretion (Sirotkin, 2010). Additionally, heat stress disturbs the ecological communities in the vagina (An et al., 2021), potentially impacting the normal function of the female reproductive tract (FRT). In this study, no significant changes were observed in plasma E_2_ and P_4_ in the Heat group. However, the plasma CAT, MDA, and T-AOC were significantly upregulated, indicating an extreme oxidative reaction in the Heat group. The unaffected plasma hormone balance may be due to the limited time of heat-stress treatment, which was consistent with the research of heat exposure for gilts (Dickson et al., 2018).

The study first reveals the bacterial composition in the rabbit’s vagina. Overall, 7 of the top 10 abundant vaginal microbiota at the phylum level were found to be common between rabbits and humans. Of them, the top 4 abundant microbiota in a rabbit’s vagina were found to be *Firmicutes*, *Actinobacteriota*, *Bacteroidota*, and *Proteobacteria*, which are also the top 4 abundant microorganisms in the human vagina. *Cyanobacteria*, *Acidobacteriota*, and *Fusobacteria* were found to be the other common microbiota. To compare, the top 4 abundant microbiota in a cow’s vagina are *Firmicutes*, *Bacteroidota*, *Proteobacteria*, and *Fusobacteria* (Galvão et al., 2019). Excluding the unidentified bacteria, *Chloroflexi* and *Verrucomicrobia* were the identified specific microbiota in rabbits’ vaginas, *Spirotomycetes* and *Planctomycetes* in humans.

The dominated genera in rabbits’ vaginas were quite different from those of humans or rats. The top 4 abundant genera in a rabbit’s vagina were identified as *Gemella*, *Corynebacteria*, *unidentified Chloroplast*, and *Porphyromonas*, while *Lactobacillus*, *Gardnerella*, *Prevotella* (or *Streptococcus*), and *Atopobium* were the top 4 dominated genera identified in human vagina (Ceccarani et al., 2019; Fu et al., 2020). The 4 most dominated genera in a cow’s vagina were *Ureaplasma*, *Anaerobiospirillum*, *Clostridium*, and *Succinivibrio* (Chen et al., 2020). These results indicate that the bacterial composition of rabbits, humans, and cows was similar at the phylum level. However, the vaginal microbiome was species specific at the genus level.

After the administration of heat stress to the Heat group, there were significant alterations in the microbiota of the rabbit vagina between the two groups. A study by Fu et al. (2020) revealed the differences in the vaginal microbiota and metabolomes between patients with RIF (RIF groups) and patients who achieved a successful clinical pregnancy in the first frozen embryo transfer cycle (normal group). Compared with the normal group, the relative abundance of *Firmicutes* significantly decreased in the RIF group, while the relative abundance of *Actinobacteria*, *Bacteroidetes*, and *Proteobacteria* increased. These changes were also observed in humans with genital infections including vulvovaginal candidiasis (VVC), Chlamydia trachomatis (CT), and BV. Surprisingly, in this study, the relative abundance of *Firmicutes* decreased in the Heat group while the relative abundance of *Actinobacteria* and *Proteobacteria* increased (Ceccarani et al., 2019). *Corynebacterium*, one of the dominant genera in rabbit vagina, increased its relative abundance in the Heat group, which is consistent with a previous study that demonstrated an increase in the abundance of *Corynebacterium* in rat vagina after heat-stress treatment (An et al., 2021). It has been suggested that *Fusobacterium* is one of the most abundant genera in cows (Santos et al., 2011; Jeon et al., 2015) and canines (Lyman et al., 2019). In the present study, *Fusobacterium* decreased four times in the Heat group. In general, the relative abundance of 18 genera was statistically different between the Heat and Control groups, of which 6 and 12 genera significantly increased and decreased (Locatelli et al., 2013), respectively. Among the increased genera, *Helcococcus* was highly prone to inflammation including human BV (Fall et al., 2018), metritis, and bacteremia (Chow and Clarridge, 2014). The diagnosis of BV may be associated with some adverse pregnancy outcomes (Nelson et al., 2007; Menard et al., 2010; Witkin, 2015). The genus *W5053* in the semen sample of bulls has been reported to be natively correlated with fertility (Cojkic et al., 2021).

On the other hand, heat stress decreased the abundance of 12 genera. Genus *Streptococcus* contains more than 100 species and was classified into groups ranging from A to W based on the antigenic reaction of the cell wall-associated carbohydrates (Lancefield and Freimer, 1966). Although a considerable group of species of *Streptococcus* is pathogenic, there is also a group of commensal and even beneficial species for animals or humans, such as *Streptococcus thermophilus* (Markakiou et al., 2020). *Streptococcus* was found to be one of the dominant genera in the vaginal samples of the giant panda, regardless of age and habitat (Yang et al., 2017; Zhang et al., 2020). The analysis of the long-term stability of urogenital microbiota of asymptomatic European women also showed the abundance of *Streptococcus*, which was found to be relevant to smoking status (Ksiezarek et al., 2021). Also, *Streptococcus* was found in rabbit feces, beneficial for digesting xylose (Borø et al., 2010). However, the abundance of *Streptococcus* has been found to increase in vagina samples of humans with genital infections (Ceccarani et al., 2019).

Similar to the sample from humans with genital infections (Ceccarani et al., 2019), HPV (Ilhan et al., 2019), or reproduction disorders (Fu et al., 2020), a decline in the abundance of *Lactobacillus* was observed in the Heat group, suspected to be susceptible to pathogenic infections. Firstly, *Lactobacillus* in the vagina can produce bacteriostatic compounds (Borges et al., 2014; Aldunate et al., 2015) against the harmful bacterium. On the other hand, their metabolite, lactic acid, can establish an acidic environment (pH ranges from 2.8 to 4.2) in the vagina (Borges et al., 2014) and attenuate pro-inflammatory responses through the production of anti-inflammatory cytokines (Hearps et al., 2017). Pretreating spermatozoa with three *Lactobacilli* decreased the level of lipid peroxidation generated by ferrous (Barbonetti et al., 2011), even though a neutral PH environment is required for the survival of spermatozoa. However, previous studies indicate that the dominance of *Lactobacillus* in the vagina is unique to humans. Although the pH in rabbits’ vaginal microenvironment is 7.2 ± 0.4 (Jacques et al., 1986), considering the ability of PH regulation and bacteriostasis of *Lactobacillus*, the decrease of *Lactobacillus* can disturb the homeostasis and increase the susceptibility to pathogenic infections in the rabbit vagina. The results of the functional analysis of the taxa for both the Heat and Control groups showed that heat stress impacted the material-and-energy-related pathways, which includes amino acid-related enzymes, glycolysis/gluconeogenesis, and starch and sucrose metabolism.

Recent studies have highlighted the connections between microbiota and metabolites in some disorders and diseases (Liu et al., 2019; Yang et al., 2019; Borgogna et al., 2020). A comprehensive analysis of the alterations of both microbiota and metabolites is beneficial for biomarker selection in some reproductive disorders. For example, Vitali et al. (2015) developed a molecular approach for BV diagnosis based on the joint analysis of the vaginal microbiome and metabolome. Multiomics analysis of immunome, metabolome, microbiome, transcriptome, and proteome of full-term pregnancy depicted comprehensive knowledge of multifaced adaptations in pregnant females, which laid a foundation for prospective diagnosis of pregnancy-related pathologies (Ghaemi et al., 2019).

The present study showed that the composition of metabolites was significantly different between the Heat and Control groups, where up to 158 metabolites increased or decreased in the rabbit vagina after heat stress exposure (*p* < 0.05). Among the top 20 differential metabolites between the two groups, the concentration of estradiol (E_2_) increased in the Heat group, and it was positively associated with linolelaidic acid (C18:2N6T), N-acetylsphingosine, N-oleoyl glycine, trans-petroselinic acid, syringic acid, and 2-(1-adamantyl)-1-morpholinoethan-1-one. Previous studies have highlighted the positive role of estrogen receptor α (ESR1) in the epithelial cells of the upper FRT, supporting fertilization or embryo development (Winuthayanon et al., 2015; Li S. et al., 2017). Both ESR1 and its ligand estrogen help build a barrier against pathogenic invasion by maintaining the epithelial thickness (Miyagawa and Iguchi, 2015) and promoting the secretion of antimicrobial peptides, cytokines, and chemokines (Salamonsen et al., 2007; Ochiel et al., 2008). Vaginal administration of estradiol has been used for the treatment of menopausal symptoms in humans. However, whether its metabolism in the human vagina is the same as in a rabbit’s vagina is unknown. Besides, no significant changes were observed in the plasma E_2_ of rabbits, implying that the hormone metabolism in the vaginal microenvironment was relatively independent of the systemic estradiol regulation. On the other hand, the associated syringic acid was found to suppress the inflammatory reaction while promoting the antioxidative biomarker expression in the mouse model of asthma (Li et al., 2019). Furthermore, syringic acid-treated human hepatoma HepG2 cells exhibited obvious morphological changes and had a higher level of reactive oxygen species and cytotoxicity. This indicates that heat stress aggravates the oxidative reaction response in the vaginal microenvironment.

Conjugated linoleic acid (CLA) is the intermediate product of polyunsaturated fatty acids (Bauman and Griinari, 2003). Linoleic acid has been suspected to promote the *in vitro* apoptosis and necrosis of lymphocytes by acting on mitochondrial depolarization and oxygen radical production (Cury-Boaventura et al., 2006). Linolelaidic acid (C18:2N6T) is one member of the CLAs that has been proved to play roles in body fat deposition (Park et al., 1997; Gaullier et al., 2004; Halade et al., 2010), tumor process (Wong et al., 1997; Ip et al., 2003; Shiraishi et al., 2010), and insulin resistance (Risérus et al., 2002, 2004). CLA displays an estrogen antagonistic effect through inhibiting ER-mediated signaling in ER-positive cancer cells (Kim et al., 2015). *In vitro* treatment with CLA inhibited Bcl-2 expression induced by estrogen in cancerous cells and promoted apoptosis (Wang et al., 2008). In this study, linolelaidic acid (C18:2N6T) was positively associated with estradiol, implying a potential balance between estrogenic and anti-estrogenic effects in vaginal ecology.

Subsequently, the study showed that differential metabolites are significantly enriched by steroid biosynthesis and endocrine and other factor-regulated calcium reabsorption pathways. The effect of heat stress on female production includes the disorder of the estrous cycle, which is often accompanied by the change in hormone levels, including E_2_ and P_4_ (Viau and Meaney, 1991). Moreover, heat stress can suppress the FSHR expression and estradiol synthesis of granulosa cells (Li H. et al., 2017). In this study, heat stress increased the level of E_2_ in the rabbit vagina, indicating an abnormal gonadal hormone-induced signaling. Dietary calcium and vitamin D insufficiency has been reported to be associated with polycystic ovary syndrome (Thys-Jacobs et al., 1999) and premenstrual syndrome (Purdue-Smithe et al., 2017). However, their metabolism in vaginal ecology is unknown. Many studies have demonstrated that heat stress can directly and indirectly affect the endocrine system and reproductive outcome (Luo et al., 2016; Li H. et al., 2017; Dickson et al., 2018). This implied heat stress affected the calcium metabolism and hormone regulation of vaginal ecology, which may impact the health and reproductive performance.

## Conclusion

Heat stress changed the composition of the microbiota and the metabolic landscape of the rabbit vagina. At the phylum level, the predominant vaginal microbiota of rabbits demonstrated significant similarity with that of humans, whereas, at the genus level, the dominance of microbiota was species specific. Heat stress decreased the relative abundance of *Firmicutes*, *Streptococcus*, and *Lactobacillus* but increased the relative abundance of *Actinobacteria*, *Proteobacteria*, *Fusobacterium*, and *W5053*, all of which have been proved to be negatively correlated with the health and reproductive functions of females in previous studies. On the other hand, a total of 158 differential metabolites in the vaginal microenvironment were identified between the Heat and Control groups. Among them, estradiol, syringic acid, and linolelaidic acid (C18:2N6T) increased in the Heat group, whereas syringic acid and linolelaidic acid (C18:2N6T) are suspected to have anti-estrogenic effects in the vaginal ecology. The KEGG analysis of differential metabolites implies that heat stress affects the hormone-induced signaling and endocrine balance of vaginal ecology. Overall, heat stress exhibited adverse effects on vaginal health through affecting the microbiome and metabolome in the vaginal ecology.

## Data Availability Statement

The datasets generated for this study has been deposited in the Sequence Read Archive (https://www.ncbi.nlm.nih.gov/sra, accession number PRJNA798555) at NCBI.

## Ethics Statement

All experimental procedure with animals were conducted with care and obey the “Guidelines for Experimental Animals” of the Ministry of Science and Technology (Beijing, China). This study was supervised and approved by the Institutional Animal Care and Use Committee (IACUC) of Sichuan Agricultural University (permit No. DKY-B2018102004).

## Author Contributions

SL, YS, XB, and LT: conceptualization. YS, LT, XB, KD, and HW: formal analysis. LT, XB, KD, XJ, and HW: resources. YS, XB, and XJ: writing—original draft preparation. YS, SL, XJ, KD, and HW: final approval. SL: supervision and funding acquisition. All authors have read and agreed to the published version of the manuscript.

## Conflict of Interest

The authors declare that the research was conducted in the absence of any commercial or financial relationships that could be construed as a potential conflict of interest.

## Publisher’s Note

All claims expressed in this article are solely those of the authors and do not necessarily represent those of their affiliated organizations, or those of the publisher, the editors and the reviewers. Any product that may be evaluated in this article, or claim that may be made by its manufacturer, is not guaranteed or endorsed by the publisher.

## Footnotes

1
http://ccb.jhu.edu/software/FLASH/

2
http://www.drive5.com/uparse/

3
http://www.arb-silva.de/

4
https://www.mzcloud.org/

5
https://www.genome.jp/kegg/pathway.html

6
https://hmdb.ca/metabolites

7
http://www.lipidmaps.org/

## Supplementary Material

Supplementary Figure 1 The annotations of all identified metabolites using the database HMDB **(A)** and LIPID **(B)** and KEGG **(C)** database. The histogram indicates the number of metabolites in the category.

Supplementary Table 1 The relative abundance of the top 10 abundant microbiota at the genus level for each individual rabbit. HS1–HS8 indicate the eight individual animals in the Heat group and CONT1–CONT8 indicate the eight individual animals in the Control group.

Supplementary Table 2 The relative concentrations of all metabolites. HS1–HS8 indicate the eight individual animals in the Heat group, and CONT1–CONT8 indicate the eight individual animals in the Control group.

## References

[B1] Aldunate M. Srbinovski D. Hearps A. C. Latham C. F. Ramsland P. A. Gugasyan R. (2015). Antimicrobial and immune modulatory effects of lactic acid and short chain fatty acids produced by vaginal microbiota associated with eubiosis and bacterial vaginosis. *Front. Physiol.* 6:164. 10.3389/fphys.2015.00164 26082720 PMC4451362

[B2] Al-Katanani Y. M. Paula-Lopes F. F. Hansen P. J. (2002). Effect of season and exposure to heat stress on oocyte competence in Holstein cows. *J. Dairy Sci.* 85 390–396. 10.3168/jds.s0022-0302(02)74086-1 11913699

[B3] An G. Zhang Y. Fan L. Chen J. Wei M. Li C. (2021). Integrative Analysis of Vaginal Microorganisms and Serum Metabolomics in Rats With Estrous Cycle Disorder Induced by Long-Term Heat Exposure Based on 16S rDNA Gene Sequencing and LC/MS-Based Metabolomics. *Front. Cell Infect. Microbiol.* 11:595716. 10.3389/fcimb.2021.595716 33738264 PMC7962411

[B4] Aroyo A. Yavin S. Arav A. Roth Z. (2007). Maternal hyperthermia disrupts developmental competence of follicle-enclosed oocytes: in vivo and ex vivo studies in mice. *Theriogenology* 67 1013–1021. 10.1016/j.theriogenology.2006.12.001 17212968

[B5] Aßhauer K. P. Wemheuer B. Daniel R. Meinicke P. (2015). Tax4Fun: predicting functional profiles from metagenomic 16S rRNA data. *Bioinformatics* 31 2882–2884. 10.1093/bioinformatics/btv287 25957349 PMC4547618

[B6] Bagath M. Krishnan G. Devaraj C. Rashamol V. P. Pragna P. Lees A. M. (2019). The impact of heat stress on the immune system in dairy cattle: a review. *Res. Vet. Sci.* 126 94–102. 10.1016/j.rvsc.2019.08.011 31445399

[B7] Barbonetti A. Cinque B. Vassallo M. R. Mineo S. Francavilla S. Cifone M. G. (2011). Effect of vaginal probiotic lactobacilli on in vitro-induced sperm lipid peroxidation and its impact on sperm motility and viability. *Fertil. Steril.* 95 2485–2488. 10.1016/j.fertnstert.2011.03.066 21497805

[B8] Bauman D. E. Griinari J. M. (2003). Nutritional regulation of milk fat synthesis. *Annu. Rev. Nutr.* 23 203–227. 10.1146/annurev.nutr.23.011702.073408 12626693

[B9] Borges S. Silva J. Teixeira P. (2014). The role of lactobacilli and probiotics in maintaining vaginal health. *Arch. Gynecol. Obstet.* 289 479–489. 10.1007/s00404-013-3064-9 24170161

[B10] Borgogna J. C. Shardell M. D. Santori E. K. Nelson T. M. Rath J. M. Glover E. D. (2020). The vaginal metabolome and microbiota of cervical HPV-positive and HPV-negative women: a cross-sectional analysis. *BJOG* 127 182–192. 31749298 10.1111/1471-0528.15981 PMC6982399

[B11] Borø S. Mccartney C. A. Snelling T. J. Worgan H. J. Mcewan N. R. (2010). Isolation of Streptococcus thoraltensis from rabbit faeces. *Curr. Microbiol.* 61 357–360. 10.1007/s00284-010-9619-0 20217090

[B12] Caporaso J. G. Kuczynski J. Stombaugh J. Bittinger K. Bushman F. D. Costello E. K. (2010). QIIME allows analysis of high-throughput community sequencing data. *Nat. Methods* 7 335–336. 10.1038/nmeth.f.303 20383131 PMC3156573

[B13] Ceccarani C. Foschi C. Parolin C. D’antuono A. Gaspari V. Consolandi C. (2019). Diversity of vaginal microbiome and metabolome during genital infections. *Sci. Rep.* 9:14095. 10.1038/s41598-019-50410-x 31575935 PMC6773718

[B14] Chen S. Y. Deng F. Zhang M. Jia X. Lai S. J. (2020). Characterization of Vaginal Microbiota Associated with Pregnancy Outcomes of Artificial Insemination in Dairy Cows. *J. Microbiol. Biotechnol.* 30 804–810. 10.4014/jmb.2002.02010 32238772 PMC9728155

[B15] Chow S. K. Clarridge J. E. III (2014). Identification and clinical significance of Helcococcus species, with description of Helcococcus seattlensis sp. nov. from a patient with urosepsis. *J. Clin. Microbiol.* 52 854–858. 10.1128/JCM.03076-13 24371247 PMC3957774

[B16] Cojkic A. Niazi A. Guo Y. Hallap T. Padrik P. Morrell J. M. (2021). Identification of Bull Semen Microbiome by 16S Sequencing and Possible Relationships with Fertility. *Microorganisms* 9:2431. 10.3390/microorganisms9122431 34946031 PMC8705814

[B17] Cury-Boaventura M. F. Gorjão R. De Lima T. M. Newsholme P. Curi R. (2006). Comparative toxicity of oleic and linoleic acid on human lymphocytes. *Life Sci.* 78 1448–1456. 10.1016/j.lfs.2005.07.038 16236329

[B18] Dickson M. J. Hager C. L. Al-Shaibi A. Thomas P. Q. Baumgard L. H. Ross J. W. (2018). Impact of heat stress during the follicular phase on porcine ovarian steroidogenic and phosphatidylinositol-3 signaling. *J. Anim. Sci.* 96 2162–2174. 10.1093/jas/sky144 29684161 PMC6095433

[B19] Edgar R. C. Haas B. J. Clemente J. C. Quince C. Knight R. (2011). UCHIME improves sensitivity and speed of chimera detection. *Bioinformatics* 27 2194–2200. 10.1093/bioinformatics/btr381 21700674 PMC3150044

[B20] El-Sheikh Ali H. Tamura Y. Sameshima H. Kitahara G. (2020). Impact of summer heat stress on the thermal environment of bovine female genital tract. *Trop. Anim. Health Prod.* 52 3449–3455. 10.1007/s11250-020-02378-4 32935322

[B21] Fall N. S. Raoult D. Sokhna C. Lagier J. C. (2018). ‘Helcococcus massiliensis’ sp. nov., a new bacterial species isolated from the vaginal sample of a woman with bacterial vaginosis living in Dielmo, Senegal. *New Microbes New Infect.* 25 27–29. 10.1016/j.nmni.2018.06.002 29997892 PMC6037904

[B22] Fu M. Zhang X. Liang Y. Lin S. Qian W. Fan S. (2020). Alterations in Vaginal Microbiota and Associated Metabolome in Women with Recurrent Implantation Failure. *mBio* 11 e03242–19. 10.1128/mBio.03242-19 32487762 PMC7267891

[B23] Galvão K. N. Higgins C. H. Zinicola M. Jeon S. J. Korzec H. Bicalho R. C. (2019). Effect of pegbovigrastim administration on the microbiome found in the vagina of cows postpartum. *J. Dairy Sci.* 102 3439–3451. 10.3168/jds.2018-15783 30799104

[B24] Gaullier J. M. Halse J. Høye K. Kristiansen K. Fagertun H. Vik H. (2004). Conjugated linoleic acid supplementation for 1 y reduces body fat mass in healthy overweight humans. *Am. J. Clin. Nutr.* 79 1118–1125. 10.1093/ajcn/79.6.1118 15159244

[B25] Ghaemi M. S. Digiulio D. B. Contrepois K. Callahan B. Ngo T. T. M. Lee-Mcmullen B. (2019). Multiomics modeling of the immunome, transcriptome, microbiome, proteome and metabolome adaptations during human pregnancy. *Bioinformatics* 35 95–103. 10.1093/bioinformatics/bty537 30561547 PMC6298056

[B26] Gupta P. Singh M. P. Goyal K. (2020). Diversity of Vaginal Microbiome in Pregnancy: deciphering the Obscurity. *Front. Public Health* 8:326. 10.3389/fpubh.2020.00326 32793540 PMC7393601

[B27] Halade G. V. Rahman M. M. Fernandes G. (2010). Differential effects of conjugated linoleic acid isomers in insulin-resistant female C57Bl/6J mice. *J. Nutr. Biochem.* 21 332–337. 10.1016/j.jnutbio.2009.01.006 19423318

[B28] Hearps A. C. Tyssen D. Srbinovski D. Bayigga L. Diaz D. J. D. Aldunate M. (2017). Vaginal lactic acid elicits an anti-inflammatory response from human cervicovaginal epithelial cells and inhibits production of pro-inflammatory mediators associated with HIV acquisition. *Mucosal Immunol.* 10 1480–1490. 10.1038/mi.2017.27 28401934

[B29] Hendricks K. E. Martins L. Hansen P. J. (2009). Consequences for the bovine embryo of being derived from a spermatozoon subjected to post-ejaculatory aging and heat shock: development to the blastocyst stage and sex ratio. *J. Reprod. Dev.* 55 69–74. 10.1262/jrd.20097 18957823

[B30] Hickey R. J. Abdo Z. Zhou X. Nemeth K. Hansmann M. Osborn T. W. III (2013). Effects of tampons and menses on the composition and diversity of vaginal microbial communities over time. *BJOG* 120 695–704. 10.1111/1471-0528.12151 23398859

[B31] Ilhan Z. E. Łaniewski P. Thomas N. Roe D. J. Chase D. M. Herbst-Kralovetz M. M. (2019). Deciphering the complex interplay between microbiota, HPV, inflammation and cancer through cervicovaginal metabolic profiling. *EBioMedicine* 44 675–690. 10.1016/j.ebiom.2019.04.028 31027917 PMC6604110

[B32] Ip M. M. Masso-Welch P. A. Ip C. (2003). Prevention of mammary cancer with conjugated linoleic acid: role of the stroma and the epithelium. *J. Mammary Gland Biol. Neoplasia* 8 103–118. 10.1023/a:1025739506536 14587866

[B33] Jacques M. Olson M. E. Crichlow A. M. Osborne A. D. Costerton J. W. (1986). The normal microflora of the female rabbit’s genital tract. *Can. J. Vet. Res.* 50 272–274. 3756680 PMC1255202

[B34] Jeon S. J. Vieira-Neto A. Gobikrushanth M. Daetz R. Mingoti R. D. Parize A. C. (2015). Uterine Microbiota Progression from Calving until Establishment of Metritis in Dairy Cows. *Appl. Environ. Microbiol.* 81 6324–6332. 10.1128/AEM.01753-15 26150453 PMC4542247

[B35] Kim J. H. Kim Y. J. Park Y. (2015). Conjugated Linoleic Acid and Postmenopausal Women’s Health. *J. Food Sci.* 80 R1137–R1143. 10.1111/1750-3841.12905 25962640

[B36] Ksiezarek M. Ugarcina-Perovic S. Rocha J. Grosso F. Peixe L. (2021). Long-term stability of the urogenital microbiota of asymptomatic European women. *BMC Microbiol.* 21:64. 10.1186/s12866-021-02123-3 33632119 PMC7905919

[B37] Lancefield R. C. Freimer E. H. (1966). Type-specific polysaccharide antigens of group B streptococci. *J. Hyg.* 64 191–203. 10.1017/s0022172400040456 5220562 PMC2134722

[B38] Langfelder P. Horvath S. (2008). WGCNA: an R package for weighted correlation network analysis. *BMC Bioinformatics* 9:559. 10.1186/1471-2105-9-559 19114008 PMC2631488

[B39] Lewis F. M. T. Bernstein K. T. Aral S. O. (2017). Vaginal Microbiome and Its Relationship to Behavior, Sexual Health, and Sexually Transmitted Diseases. *Obstet. Gynecol.* 129 643–654. 10.1097/AOG.0000000000001932 28277350 PMC6743080

[B40] Li H. Guo S. Cai L. Ma W. Shi Z. (2017). Lipopolysaccharide and heat stress impair the estradiol biosynthesis in granulosa cells via increase of HSP70 and inhibition of smad3 phosphorylation and nuclear translocation. *Cell Signal.* 30 130–141. 10.1016/j.cellsig.2016.12.004 27940052

[B41] Li S. O’neill S. R. Zhang Y. Holtzman M. J. Takemaru K. I. Korach K. S. (2017). Estrogen receptor α is required for oviductal transport of embryos. *FASEB J.* 31 1595–1607. 10.1096/fj.201601128R 28082352 PMC5349796

[B42] Li Y. Zhang L. Wang X. Wu W. Qin R. (2019). Effect of Syringic acid on antioxidant biomarkers and associated inflammatory markers in mice model of asthma. *Drug Dev. Res.* 80 253–261. 10.1002/ddr.21487 30474283

[B43] Liu F. Sun Z. Hu P. Tian Q. Xu Z. Li Z. (2019). Determining the protective effects of Yin-Chen-Hao Tang against acute liver injury induced by carbon tetrachloride using 16S rRNA gene sequencing and LC/MS-based metabolomics. *J. Pharm. Biomed. Anal.* 174 567–577. 10.1016/j.jpba.2019.06.028 31261038

[B44] Locatelli C. Scaccabarozzi L. Pisoni G. Bronzo V. Casula A. Testa F. (2013). Helcococcus kunzii and Helcococcus ovis isolated in dairy cows with puerperal metritis. *J. Gen. Appl. Microbiol.* 59 371–374. 10.2323/jgam.59.371 24201149

[B45] Luo M. Li L. Xiao C. Sun Y. Wang G. L. (2016). Heat stress impairs mice granulosa cell function by diminishing steroids production and inducing apoptosis. *Mol. Cell. Biochem.* 412 81–90. 10.1007/s11010-015-2610-0 26602771

[B46] Lyman C. C. Holyoak G. R. Meinkoth K. Wieneke X. Chillemi K. A. Desilva U. (2019). Canine endometrial and vaginal microbiomes reveal distinct and complex ecosystems. *PLoS One* 14:e0210157. 10.1371/journal.pone.0210157 30615657 PMC6322750

[B47] Magoč T. Salzberg S. L. (2011). FLASH: fast length adjustment of short reads to improve genome assemblies. *Bioinformatics* 27 2957–2963. 10.1093/bioinformatics/btr507 21903629 PMC3198573

[B48] Marai I. F. Ayyat M. S. Abd El-Monem U. M. (2001). Growth performance and reproductive traits at first parity of New Zealand white female rabbits as affected by heat stress and its alleviation under Egyptian conditions. *Trop. Anim. Health Prod.* 33 451–462. 10.1023/a:1012772311177 11770200

[B49] Marco-Jiménez F. Naturil-Alfonso C. Peñaranda D. S. Jiménez-Trigos E. García-Diego F. J. Vicente J. S. (2013). Maternal exposure to high temperatures disrupts OCT4 mRNA expression of rabbit pre-implantation embryos and endometrial tissue. *Reprod. Domest. Anim.* 48 429–434. 10.1111/rda.12092 23043275

[B50] Markakiou S. Gaspar P. Johansen E. Zeidan A. A. Neves A. R. (2020). Harnessing the metabolic potential of *Streptococcus thermophilus* for new biotechnological applications. *Curr. Opin. Biotechnol.* 61 142–152. 10.1016/j.copbio.2019.12.019 31945498

[B51] Menard J. P. Mazouni C. Salem-Cherif I. Fenollar F. Raoult D. Boubli L. (2010). High vaginal concentrations of Atopobium vaginae and Gardnerella vaginalis in women undergoing preterm labor. *Obstet. Gynecol.* 115 134–140. 10.1097/AOG.0b013e3181c391d7 20027045

[B52] Miyagawa S. Iguchi T. (2015). Epithelial estrogen receptor 1 intrinsically mediates squamous differentiation in the mouse vagina. *Proc. Natl. Acad. Sci. U. S. A.* 112 12986–12991. 10.1073/pnas.1513550112 26438838 PMC4620905

[B53] Mutwedu V. B. Nyongesa A. W. Oduma J. A. Kitaa J. M. Mbaria J. M. (2021). Thermal stress causes oxidative stress and physiological changes in female rabbits. *J. Therm. Biol.* 95:102780. 10.1016/j.jtherbio.2020.102780 33454048

[B54] Nabenishi H. Ohta H. Nishimoto T. Morita T. Ashizawa K. Tsuzuki Y. (2011). Effect of the temperature-humidity index on body temperature and conception rate of lactating dairy cows in southwestern Japan. *J. Reprod. Dev.* 57 450–456. 10.1262/jrd.10-135t 21478652

[B55] Nelson D. B. Bellamy S. Nachamkin I. Ness R. B. Macones G. A. Allen-Taylor L. (2007). First trimester bacterial vaginosis, individual microorganism levels, and risk of second trimester pregnancy loss among urban women. *Fertil. Steril.* 88 1396–1403. 10.1016/j.fertnstert.2007.01.035 17434499 PMC2094106

[B56] Ochiel D. O. Fahey J. V. Ghosh M. Haddad S. N. Wira C. R. (2008). Innate Immunity in the Female Reproductive Tract: role of Sex Hormones in Regulating Uterine Epithelial Cell Protection Against Pathogens. *Curr. Womens Health Rev.* 4 102–117. 10.2174/157340408784246395 19644567 PMC2717724

[B57] Onderdonk A. B. Delaney M. L. Fichorova R. N. (2016). The Human Microbiome during Bacterial Vaginosis. *Clin. Microbiol. Rev.* 29 223–238. 10.1128/CMR.00075-15 26864580 PMC4786887

[B58] Oshima Y. Coutts R. D. Badlani N. M. Healey R. M. Kubo T. Amiel D. (2011). Effect of light-emitting diode (LED) therapy on the development of osteoarthritis (OA) in a rabbit model. *Biomed. Pharmacother.* 65 224–229. 10.1016/j.biopha.2011.02.011 21658899

[B59] Ozawa M. Hirabayashi M. Kanai Y. (2002). Developmental competence and oxidative state of mouse zygotes heat-stressed maternally or in vitro. *Reproduction* 124 683–689. 10.1530/rep.0.1240683 12417007

[B60] Park Y. Albright K. J. Liu W. Storkson J. M. Cook M. E. Pariza M. W. (1997). Effect of conjugated linoleic acid on body composition in mice. *Lipids* 32 853–858. 9270977 10.1007/s11745-997-0109-x

[B61] Paul C. Murray A. A. Spears N. Saunders P. T. (2008). A single, mild, transient scrotal heat stress causes DNA damage, subfertility and impairs formation of blastocysts in mice. *Reproduction* 136 73–84. 10.1530/REP-08-0036 18390691

[B62] Purdue-Smithe A. C. Whitcomb B. W. Szegda K. L. Boutot M. E. Manson J. E. Hankinson S. E. (2017). Vitamin D and calcium intake and risk of early menopause. *Am. J. Clin. Nutr.* 105 1493–1501. 10.3945/ajcn.116.145607 28490509 PMC5445672

[B63] Quast C. Pruesse E. Yilmaz P. Gerken J. Schweer T. Yarza P. (2013). The SILVA ribosomal RNA gene database project: improved data processing and web-based tools. *Nucleic Acids Res.* 41 D590–D596. 10.1093/nar/gks1219 23193283 PMC3531112

[B64] Risérus U. Arner P. Brismar K. Vessby B. (2002). Treatment with dietary trans10cis12 conjugated linoleic acid causes isomer-specific insulin resistance in obese men with the metabolic syndrome. *Diabetes Care* 25 1516–1521. 10.2337/diacare.25.9.1516 12196420

[B65] Risérus U. Vessby B. Arnlöv J. Basu S. (2004). Effects of cis-9,trans-11 conjugated linoleic acid supplementation on insulin sensitivity, lipid peroxidation, and proinflammatory markers in obese men. *Am. J. Clin. Nutr.* 80 279–283. 10.1093/ajcn/80.2.279 15277146

[B66] Salamonsen L. A. Hannan N. J. Dimitriadis E. (2007). Cytokines and chemokines during human embryo implantation: roles in implantation and early placentation. *Semin. Reprod. Med.* 25 437–444. 10.1055/s-2007-991041 17960528

[B67] Santos T. M. Gilbert R. O. Bicalho R. C. (2011). Metagenomic analysis of the uterine bacterial microbiota in healthy and metritic postpartum dairy cows. *J. Dairy Sci.* 94 291–302. 10.3168/jds.2010-3668 21183039

[B68] Shiraishi R. Iwakiri R. Fujise T. Kuroki T. Kakimoto T. Takashima T. (2010). Conjugated linoleic acid suppresses colon carcinogenesis in azoxymethane-pretreated rats with long-term feeding of diet containing beef tallow. *J. Gastroenterol.* 45 625–635. 10.1007/s00535-010-0206-8 20143104

[B69] Sirotkin A. V. (2010). Effect of two types of stress (heat shock/high temperature and malnutrition/serum deprivation) on porcine ovarian cell functions and their response to hormones. *J. Exp. Biol.* 213 2125–2130. 10.1242/jeb.040626 20511527

[B70] Thys-Jacobs S. Donovan D. Papadopoulos A. Sarrel P. Bilezikian J. P. (1999). Vitamin D and calcium dysregulation in the polycystic ovarian syndrome. *Steroids* 64 430–435. 10.1016/s0039-128x(99)00012-4 10433180

[B71] Ulberg L. C. Burfening P. J. (1967). Embryo death resulting from adverse environment on spermatozoa or ova. *J. Anim. Sci.* 26 571–577. 10.2527/jas1967.263571x 6068502

[B72] Viau V. Meaney M. J. (1991). Variations in the hypothalamic-pituitary-adrenal response to stress during the estrous cycle in the rat. *Endocrinology* 129 2503–2511. 10.1210/endo-129-5-2503 1657578

[B73] Vitali B. Cruciani F. Picone G. Parolin C. Donders G. Laghi L. (2015). Vaginal microbiome and metabolome highlight specific signatures of bacterial vaginosis. *Eur. J. Clin. Microbiol. Infect. Dis.* 34 2367–2376. 10.1007/s10096-015-2490-y 26385347

[B74] Wang L. S. Huang Y. W. Liu S. Yan P. Lin Y. C. (2008). Conjugated linoleic acid induces apoptosis through estrogen receptor alpha in human breast tissue. *BMC Cancer* 8:208. 10.1186/1471-2407-8-208 18652667 PMC2517598

[B75] Wen B. Mei Z. Zeng C. Liu S. (2017). metaX: a flexible and comprehensive software for processing metabolomics data. *BMC Bioinformatics* 18:183. 10.1186/s12859-017-1579-y 28327092 PMC5361702

[B76] Wen C. Li S. Wang J. Zhu Y. Zong X. Wang Y. (2021). Heat Stress Alters the Intestinal Microbiota and Metabolomic Profiles in Mice. *Front. Microbiol.* 12:706772. 10.3389/fmicb.2021.706772 34512584 PMC8430895

[B77] Wickham H. (2009). *Ggplot2: Elegant Graphics for Data Analysis.* New York, NY: Springer.

[B78] Winuthayanon W. Bernhardt M. L. Padilla-Banks E. Myers P. H. Edin M. L. Lih F. B. (2015). Oviductal estrogen receptor α signaling prevents protease-mediated embryo death. *Elife* 4:e10453. 10.7554/eLife.10453 26623518 PMC4718728

[B79] Witkin S. S. (2015). The vaginal microbiome, vaginal anti-microbial defence mechanisms and the clinical challenge of reducing infection-related preterm birth. *BJOG* 122 213–218. 10.1111/1471-0528.13115 25316066

[B80] Wong M. W. Chew B. P. Wong T. S. Hosick H. L. Boylston T. D. Shultz T. D. (1997). Effects of dietary conjugated linoleic acid on lymphocyte function and growth of mammary tumors in mice. *Anticancer Res.* 17 987–993. 9137439

[B81] Xiong Y. Yi H. Wu Q. Jiang Z. Wang L. (2020). Effects of acute heat stress on intestinal microbiota in grow-finishing pigs, and associations with feed intake and serum profile. *J. Appl. Microbiol.* 128 840–852. 10.1111/jam.14504 31671233

[B82] Yang X. Cheng G. Li C. Yang J. Li J. Chen D. (2017). The normal vaginal and uterine bacterial microbiome in giant pandas (*Ailuropoda melanoleuca*). *Microbiol. Res.* 199 1–9. 10.1016/j.micres.2017.01.003 28454704

[B83] Yang Y. Misra B. B. Liang L. Bi D. Weng W. Wu W. (2019). Integrated microbiome and metabolome analysis reveals a novel interplay between commensal bacteria and metabolites in colorectal cancer. *Theranostics* 9 4101–4114. 10.7150/thno.35186 31281534 PMC6592169

[B84] Zhang L. Li C. Zhai Y. Feng L. Bai K. Zhang Z. (2020). Analysis of the vaginal microbiome of giant pandas using metagenomics sequencing. *Microbiologyopen* 9:e1131. 10.1002/mbo3.1131 33205903 PMC7755806

[B85] Zhu L. Liao R. Wu N. Zhu G. Yang C. (2019). Heat stress mediates changes in fecal microbiome and functional pathways of laying hens. *Appl. Microbiol. Biotechnol.* 103 461–472. 10.1007/s00253-018-9465-8 30368579

