# Supplementary material for: Heat Stress Altered the Vaginal Microbiome and Metabolome in Rabbits
Source: Front Microbiol. 2022 Apr 14;13:813622. doi: 10.3389/fmicb.2022.813622 (PMC9048824; doi:10.3389/fmicb.2022.813622)
Supplement: Supplementary file 1 [file Presentation_1.pdf]

Supplementary figure and table legend

Figure 1 The annotations of all identified metabolites using the database HMDB (A) and LIPID (B) and KEGG (C) database. The histogram indicates the number of metabolites in the category.

Table 1 The relative abundance of top 10 abundant microbiota at the genus level for each individual rabbit. HS1-HS8 indicates the 8 individual animals in the Heat group and CONT1-CONT8 indicates the 8 individual animals in the Control group.

Table 2 The relative concentrations of all metabolites. HS1-HS8 indicates the 8 individual animals in the Heat group and CONT1-CONT8 indicates the 8 individual animals in the Control group.
