# Supplementary figures and images for: Heat Stress Altered the Vaginal Microbiome and Metabolome in Rabbits
Source: Front Microbiol. 2022 Apr 14;13:813622. doi: 10.3389/fmicb.2022.813622 (PMC9048824; doi:10.3389/fmicb.2022.813622)

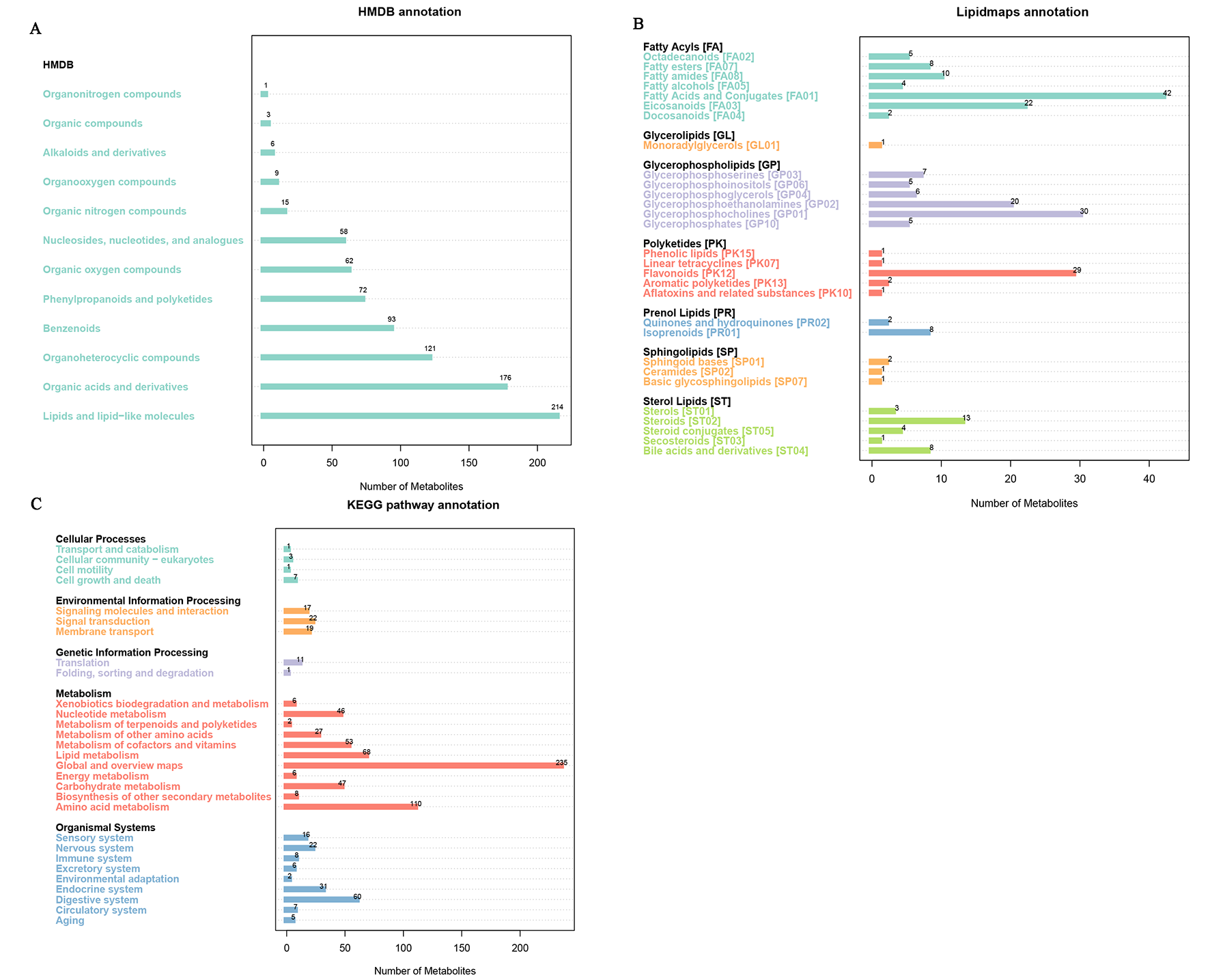

Supplement: Supplementary Figure 1 — The annotations of all identified metabolites using the database HMDB (A) and LIPID (B) and KEGG (C) database. The histogram indicates the number of metabolites in the category. [file Image_1.TIF]
